# Detection and Quantification of Fluorescent Cell Clusters in Cryo-Imaging

**DOI:** 10.1155/2012/698413

**Published:** 2012-03-18

**Authors:** Grant J. Steyer, Feng Dong, Lehar Kanodia, Debashish Roy, Marc Penn, David L. Wilson

**Affiliations:** ^1^Department of Biomedical Engineering, Case Western Reserve University, 10900 Euclid Avenue, Cleveland, OH 44106, USA; ^2^Department of Cardiology, The Cleveland Clinic, 9500 Euclid Avenue, Cleveland, OH 44195, USA; ^3^Department of Radiology, Case Western Reserve University, 10900 Euclid Avenue, Cleveland, OH 44106, USA

## Abstract

We developed and evaluated an algorithm for enumerating fluorescently labeled cells (e.g., stem and cancer cells) in mouse-sized, microscopic-resolution, cryo-image volumes. Fluorescent cell clusters were detected, segmented, and then fit with a model which incorporated a priori information about cell size, shape, and intensity. The robust algorithm performed well in phantom and tissue imaging tests, including accurate (<2% error) counting of cells in mouse. Preliminary experiments demonstrate that cryo-imaging and software can uniquely analyze delivery, homing to an organ and tissue distribution of stem cell therapeutics.

## 1. Introduction

Stem cell therapies are promising treatments for many diseases, and preclinical trials in ischemic heart disease show enhanced recovery of function [1, 2]. While mechanisms for homing are being defined, exogenous stem cell therapies are being implemented in clinical trials, despite a limited understanding of mechanisms. Due to limitations of current methods such as histology, QPCR, and *in vivo* imaging, one cannot reliably determine efficiency and dose response of exogenous stem cell therapy. In order to determine dose response of stem cell treatments, imaging must be performed with single cell sensitivity and resolution over large regions of an entire specimen. The utility of imaging small numbers of cells over a large specimen is not limited to stem cell research, but is needed in immune response, cancer, and many other fields of research [2].

Cryo-imaging, as defined below, provides single cell resolution and sensitivity over an entire specimen, which is not possible with *in vivo*, small animal imaging systems such as CT, MRI, PET, SPECT, whole animal fluorescence, or bioluminescence. Optical imaging modalities such as intravital imaging [3] do not offer the field of view (FOV) or depth of field of cryo-imaging. By imaging with high resolution and sensitivity, it is possible to identify fluorescently-labeled single cells or cell clusters within a mouse. Once cells are identified, cell locations can be mapped relative to the tissue anatomy in the high contrast, 3D cryo-image color volumes.

Cryo-imaging consists of a modified, bright-field/fluorescence microscope, a robotic imaging system positioner, a customized, whole mouse motorized cryomicrotome, control system, and analysis/visualization software [4]. By alternately sectioning and imaging the specimen, the system acquires brightfield color and fluorescent image volumes, providing micron-scale resolution, detailed views of anatomy, and cells labeled with fluorescent reporter genes or exogenous fluorophores [5–8].

 Once images have been collected of an entire mouse at sufficient resolution to detect individual fluorescently labeled cells, methods must be developed to detect and segment fluorescently labeled cell clusters and quantify the number of cells per cluster. A cryo-image volume consists of tens of thousands of images and over 80 GB, necessitating the need for an automated approach. To put our task in perspective, finding a one-voxel cell in a whole mouse cryo-image volume is equivalent to finding one voxel in 23 × 10^9^ voxels, or about one needle in 1900 haystacks. Typical cellular quantification algorithms range in sophistication from simple manual dot counting, to automated deformable models [9] and neural network-based segmentation [10]. The latter methods require images of sufficient resolution, such that the cell boundary is distinguishable. In order to limit the number of images required to image an entire mouse, cryo-imaging is performed with a 15.6 *μ*m pixel size, as compared to the smallest available pixel size of 1.2 *μ*m. At this resolution, cells are approximately the size of a single pixel, making it impossible to resolve cell boundaries in large clusters of cells. For this reason, it is necessary to utilize quantification algorithms that do not rely on high resolution to perform quantification.

There are reports of model-based segmentation and quantification algorithms when the size and shape of cells varies greatly and the boundary between cells is indistinguishable due to low resolution or contrast [11]. The cell model is used to incorporate prior information about cell size, shape, and intensity, as well as the transfer function of the imaging system [12]. A common model of cells is a Gaussian [11–13]. Cells are approximately the size of a pixel, which appears as a single bright point. This point is then blurred during imaging due to pixilation, the transfer function of the imaging system, and light scattering by the tissue. By modeling a cell as a Gaussian, we are accounting for the spherical shape of the cells, as well as blurring due to the imaging process. Currently, model-based algorithms have been used for segmentation of cells, cell clusters, and subcellular structures [11–13]. Possible cells or cell clusters are identified and fit to a range of Gaussians with varying *σ*'s [11, 13] or an optimal *σ* was estimated through optimization [12]. Noordmans and Smeulders attempted to isolate overlapping spots by subtracting a preliminary fit of one of the spots from the image of the overlapping spots [13]. Two spots that overlapped to a large extent were erroneously counted as single cells and estimates of the individual spot sizes were inaccurate. To our knowledge, no attempt has been reported in the literature to separate more than two cells using model-based algorithms.

In this paper, we develop and evaluate a model-based algorithm for enumerating 1000's of fluorescently labeled cells in a cryo-image volume. The algorithm includes detection, segmentation, and quantification steps. Knowledge-based image processing is performed to remove false positives (FP's), where rules are created based on known properties of cell clusters. In experiments, we image homogeneous phantoms containing fluorescent microspheres or quantum dot-labeled mouse mesenchymal stem cells MSC's. Results are validated against high-resolution imaging and against human interpretation. In sections that follow, we develop the algorithms, describe the software and parameter estimation, validate our method, and process cryo-image volumes.

## 2. Algorithm

We divide cell processing into three major steps: preprocessing, detection/segmentation of cell clusters, and estimation of the number of cells per cluster. Algorithms are described for the case of red quantum dot-labeled cells. Later, we describe modifications required for cells labeled with fluorescent proteins.

### 2.1. Preprocess Images

We tile and align cryo-images using semiautomated gray-scale registration algorithms previously described [14]. Subsurface fluorescence can contribute to a given image, but we have developed a “next-image” processing algorithm which isolates the fluorescence within a given section [15]. Briefly, to account for attenuation and scatter in tissue, we blur and attenuate the next image in the stack and subtract it from the current image. For optimal processing with high microscope resolution and thin sections, we estimate parameters from images using an optimization algorithm [15]. Careful fine tuning of tissue-specific parameters is less critical at lower microscope resolution and thicker sections. For example, in our application experiment with 40 *μ*m sections, quantum dot-labeled stem cells appear as a single pixel and are visible over at most 2-3 images at greatly diminished intensity. In such experiments, the subsurface fluorescence intensity is much lower, *≈*15–20% of the intensity of fluorescent cells at the surface, and any subsurface fluorescence not removed by next-image processing will be too dim to be confused as a fluorescently labeled cell. Next-image processing greatly reduces any subsurface fluorescence from labeled cells as well as general background autofluorescence.

 We also process images to remove background autofluorescence. This process is necessary to get reliable values of cluster intensity for subsequent processing. Morphological reconstruction [16] is performed to determine the autofluorescent background. We found that morphological reconstruction gave better results than morphological grayscale opening, as it reduced artifacts, such as “blotching,” where regions of pixels have unnaturally uniform intensity [17] and corrected the background intensity under the clusters. Morphological reconstruction takes as an input a marker image and the original image, sometimes called a mask image in the literature. In order to determine the autofluorescent background, a marker image is chosen with fluorescent clusters removed. This marker image is repeatedly dilated until the contour of the marker image fits under the original image. This results in a reconstructed image that contains only the background autofluorescence. In our application, we process each color channel separately, with the original 2D color fluorescent image as the original image. In order to determine the autofluorescent background, all fluorescent cell clusters should be removed from the marker image. We determine the marker image by performing morphological grayscale erosion on the original image, removing all possible cell clusters. To make certain that all fluorescent clusters are removed during erosion, the structuring element must be larger than all fluorescent clusters present in the image. Erosion was performed with a disk structuring element of radius 10 pixels, which was experimentally determined to remove all clusters. Following morphological reconstruction, the reconstructed image contains only background; all areas of local maxima (cell clusters and FP's) are removed. By subtracting this reconstructed background image from the original image, we are left with an image containing fluorescent clusters as well as some FP's.

For cells containing red quantum dots, we compute the ratio of the red to green fluorescence digital image intensities, *I*_*R*_/*I*_*G*_. Fluorescence images excited in the range of 390–465 nm contain predominately green autofluorescence, giving *I*_*R*_/*I*_*G*_ ≈ 0.4. Very few autofluorescent pixels are more red than green. By comparison, cells labeled with red (625 nm) quantum dots are much redder than green, giving *I*_*R*_/*I*_*G*_ ≈ 8.0. By thresholding the ratio image *I*_*R*_/*I*_*G*_ with a threshold value *T*_*R*/*G*_ (1), we are able to remove nearly all effects of autofluorescence. The threshold *T*_*R*/*G*_ is interactively determined by a user to conservatively include all cells and possibly some FP's, which will be later pruned away. The thresholded ratio image is multiplied by *I*_*R*_ (1), the red channel intensity of the original image, creating a grayscale image, as below, where *binary* is the creation of a binary image using the enclosed operation.

(1) $$I_{\text{Gray}}=I_{R}*\text{binary}\left(\frac{I_{R}}{I_{G}}\;>T_{R/G}\right).$$

### 2.2. Detect/Segment

To segment fluorescent clusters hysteresis thresholding was performed on *I*_Gray_. Hysteresis thresholding first segments *I*_Gray_ by a high threshold (*T*_*H*_), leaving only the brightest pixels in each cluster of cells [18]. A low threshold (*T*_*L*_) is then applied, which includes all pixels in the fluorescent clusters, as well as FP's due to autofluorescence. Pixels selected by the low threshold are retained if they are connected to a pixel selected by the high threshold. Low and high thresholds are interactively determined. The user selects a high threshold, such that all clusters contain at least one thresholded pixel. The high threshold should be chosen to include all cells. For this reason, the high threshold is typically determined from an image of cells in culture. The high threshold is determined based on the maximum intensity of each cell cluster in culture, described below in  (2)

(2) $$T_{H}=.6*\min\left[\forall n:C_{\max}\left(n\right)\right],$$

where *C*_max⁡_(*n*) is the maximum intensity of a cell cluster *n* in culture. The high threshold can be increased to reduce the number of false positives. However, this is often avoided to ensure that all cell clusters are segmented. The low threshold is typically selected to exclude the autofluorescent background. The low threshold is determined based on the mean and standard deviation of the background as described below in (3)

(3) $$T_{L}=\mu_{\text{img}}+c*\sigma_{\text{img}},$$

where *μ*_img_ is the mean value of the digital image intensity values, *σ*_img_ is the standard deviation, and *c* is a constant. From experimental analysis, a value of *c* = 1.4 or greater was found to reduce the inclusion of autofluorescence, while including the entirety of all cell clusters. A user may elect to increase the low threshold to reduce the inclusion of dim autofluorescent pixels.

Connected component analysis (CCA) is performed to uniquely label each segmented fluorescent cluster [19]. From each uniquely labeled cluster, cluster features consisting of volume, integrated intensity, bounding box, integrated intensity to volume ratio, and center of mass are determined. These features are later used to reject FP clusters and to initiate model-based analysis.

In general, the above thresholds are set to “over call” fluorescent clusters, ensuring that all cells are identified. This may include FP's, which are removed through knowledge-based processing. Since cells are well separated prior to injection and since we are focusing on stem cell studies over relatively short-time periods (1 hour to 6 days) with minimal cell division, cell clusters are typically small with many one and two cell clusters and very few with as many as 15 cells in a cluster. The vast majority of FPs due to autofluorescence can be removed by setting a maximum cluster size. Very large clusters due to bright autofluorescent regions of the intestine, bone, and stomach are easily removed in this way. Small autofluorescent structures (e.g., remnants of the intestine and small bones) may not be removed by the maximum size rule. However, we have determined that by setting a minimum and maximum intensity-to-volume ratio, we can easily remove such regions. In this way, it is possible to remove small structures that are too bright or too dim to be labeled cells. Rules are (1) fluorescent clusters with a volume (*C*_*V*_) larger than a user defined threshold (*T*_*V*_) are classified as FP's (*C*_*V*_ > *T*_*V*_); (2) fluorescent clusters with an integrated intensity (∫*C*_*i*_) to volume (*C*_*V*_) ratio outside of a specified range (*T*_*R*,*L*_: low-ratio threshold, *T*_*R*,*H*_: high-ratio threshold) are classified as FP's (∫*C*_*i*_/*C*_*V*_ < *T*_*R*,*L*_, ∫*C*_*i*_/*C*_*V*_ > *T*_*R*,*H*_). Conservative thresholds are used to guard against the possibility of mistakenly removing cells. Additional FP removal is performed following model-based quantification to further reduce FP's.

### 2.3. Estimate the Number of Cells/Cluster

The first step in our model-based analysis is to determine average parameters for a single cell. Histograms of cluster features (volumes and integrated intensities) give qualitative information about the number of cells per cluster. The largest peaks in the histograms correspond to single cells. Because the average stem cell diameter is 10–15 *μ*m, 3-4 times smaller than the slice thickness (40 *μ*m), the vast majority of single cells should be contained within a single slice. Only fluorescent clusters contained within a single 2D cryo-image are assumed to be single cells. Each single cell is fit to a Gaussian with free parameters; *σ*, integrated intensity (*I*_*t*_), and the center of the Gaussian (*x*_*c*_ and *y*_*c*_). Parameters are estimated using a least squares error objective function and Levenberg-Marquardt or Nelder-Mead nonlinear optimizer [20, 21]. Model parameters are averaged to obtain the model for a single cell.

Model-based analysis is performed to estimate the number of cells per cluster for all detected clusters in the volume. For any given cluster, we first estimate the number of cells in a cluster (*N*_*i*_) as shown in  (4):

(4) $$N_{i}=\frac{\int C_{i}}{I_{t}},$$

where ∫*C*_*i*_ is the integrated intensity of cluster *i* and *I*_*t*_ is the total intensity of the model single cell. A range of integer numbers of cells around this estimate is used to determine the range for fitting the cluster. The range of number of cells tested is given by  (5):

(5) $$N_{i}\pm \max\left(2,N_{i}*.30\right).$$

For each integer number of cells tested, *n*, the optimal placement of *n* model cells that most closely resembles the 3D image of the cluster is determined. For each model cell there are free parameters *x*_*c*_, *y*_*c*_, and *z*_*c*_, where *x*_*c*_, *y*_*c*_, and *z*_*c*_ are real valued parameters setting the center of the cell and *x*_*c*_ and *y*_*c*_ set the location of a 2D Gaussian. Recall that we use thick sections (40 *μ*m) as compared to the resolution in the *xy*-plane (8.8–15.6 *μ*m) and that there is a potential of sectioning a single cell. Hence, *z*_*c*_ is treated differently from *x*_*c*_ and *y*_*c*_. The model cell is set at a “height” *z*_*c*_. To account for sectioned cells, *z*_*c*_ specifies the percentage of a cell contained in neighboring sections. The division of the cell between adjoining slices is used to divide the total intensity (*I*_*t*_) between the slices. The center of two cells may not be closer than *D*_min⁡_, the approximate diameter of a single cell. This prevents multiple cells from occupying the same 3D space. Optimal placement of the *n* model cells is determined through minimization of an objective function using the nonlinear optimizer. The objective function, *F*_obj_, is least square error, as given below

(6) $$F_{\text{obj}}=\sqrt{\overset{m}{\sum }{\left(I_{\text{Gray}}-\overset{n}{\sum }I_{\text{gauss}}\right)}^{2}},$$

where *I*_Gray_ is the grayscale fluorescent image; *I*_gauss_ is a single model cell with input parameters *x*_*c*_, *y*_*c*_, *z*_*c*_, *I*_*t*_, and *σ*; *m* is the number of pixels in the cluster; *n* is the total number of model cells. The number of cells *n* that best matches the 3D cluster image, as determined through least square error, is designated as the number of cells in the cluster. Please note that (4) is simply used to obtain an initial guess and that (6) is the working equation for determining the actual number of cells in a cluster. The method assumes that cells have a fixed size and intensity.

### 2.4. Interactive Determination of Processing Parameters

Optimal algorithm parameters will depend at least upon spectral characteristics of the fluorophore, cell size, and intensity. At this time, parameters are interactively selected by a user. We have found that similar experiments can be analyzed using the same parameters. In our interface, a series of 2D images from a selected cryo-image volume are displayed for the user to interact with and determine the threshold values by examining the affect of changing *T*_*R*/*G*_, *T*_*H*_, and *T*_*L*_ on the number of clusters and FP's included, as well as which pixels are included within a cluster. As described above, a threshold *T*_*R*/*G*_ is required to reduce the color fluorescent image volume to the grayscale image volume *I*_Gray_. Two additional thresholds *T*_*H*_ and *T*_*L*_ are required to perform hysteresis thresholding on *I*_Gray_. Initial guesses for *T*_*R*/*G*_, *T*_*H*_, and *T*_*L*_ are made by the user based on images of cells in the dish as described above, prior experiments, or observed cell intensities in the displayed images. Initial guesses are made low to include all cells and FP's. Using these original threshold values, the original image is processed to create the hysteresis thresholded image *I*_Hyst_. In separate windows, the original image and *I*_Hyst_ are displayed. Zoomed in views of possible cell locations of the original color cryo-image are displayed along with statistical measurements of the object and the necessary *T*_*R*/*G*_, *T*_*H*_, and *T*_*L*_ to include or reject the object. Due to the brightness of labeled cells, the repetitive features of autofluorescence in different tissues, and the known tissue location of the object, a user is able to confirm or reject that a given object is a cell. User predictions can be compared to multispectral images of the same location to confirm that a given object is indeed a labeled cell. Once parameters are set, we can process large tissue regions at will. Pseudocode for this entire process is shown in Table 1.

## 3. Experimental Methods

### 3.1. Instrument

The Case whole mouse cryo-imaging test bed system has been previously described [4, 15]. Briefly, it consists of a modified large section cryo-microtome, XYZ robotic positioner carrying an imaging system which consists of a stereo microscope, low-light digital camera, and brightfield and fluorescent light sources. The cryo-imaging system is controlled by a control computer running Labview (National Instruments, Austin, TX). The stereo microscope uses multiple objectives (0.036 NA and 0.11 NA) and zoom settings (7–90x), and the FOV can be varied to cover an entire mouse or down to a small organ with a maximum in-plane resolution of 1.2 *μ*m. To enable very high-resolution imaging over a large FOV, the XYZ robotic positioner moves the imaging system over the entire specimen, creating a micronscale-tiled image acquisition. Once collected, images were automatically aligned to correct for small (micron-scale) misalignments using registration software and corrected by hand where necessary. Preprocessing and cell quantification software were written in Matlab (Mathworks, Natick, MA). Visualization was done within Amira using specialized functions of our own design [14]. Processing was done on imaging workstations having with as much as 32 GB RAM and Intel Xeon 8-core 3.00 GHz processor running Windows XP 64 bit.

### 3.2. Phantom Experiments

To develop the quantification algorithm and to test its accuracy, two phantoms were created. First, we embedded 15 *μ*m diameter green fluorescent microspheres (FluoSpheres, Invitrogen, Carlsbad, CA) within optimal cutting temperature compound (OCT) (Tissue Tek, Ted Pella, Inc., Redding, CA). The intensity and size of fluorescent microspheres varies by less than 5% and provides an ideal alternative to live cells. To further validate our method, mouse mesenchymal stem cells (MSC's) were labeled with quantum dots (Qtracker 625 Cell Labeling Kit, Invitrogen, Carlsbad, CA) and embedded within OCT.

Microspheres were snap frozen in liquid nitrogen and cryo-imaged with a section thickness of 40 *μ*m. Phantoms were imaged at normal operating resolution (7x, 15.6 *μ*m pixels) and high resolution (63x, 1.6 *μ*m pixels for microspheres and 20x, 5.4 *μ*m pixels for cells). At high resolution, the number of microspheres/cells per cluster was visually apparent, while the number of microspheres/cells per cluster was not distinguishable at the lower resolution. Model based processing was performed on the low-resolution images. In experiments, the computer-estimated number of microspheres/cells at the normal operating resolution was compared to the number counted by an expert reader using high-resolution images. The expert reader was not the algorithm developer. To determine any variability in the manual analysis, the research team spot-checked results of the expert reader. In all cases, consensus determined that the reader unequivocally determined counts per cluster using the high-resolution images. Since intraobserver variability in manual reading would be negligible, we deemed it unnecessary to assess variability of our “gold standard” using multiple readers.

### 3.3. Animal Preparation and LAD Ligation

All animal protocols were approved by the Animal Research Committee and all animals were housed in the AAALAC animal facility of the Cleveland Clinic. All mice used in this study were C57BL/6J male mice obtained from the Jackson Laboratory (Bar Harbor, ME) at 4–6 weeks of age. Anterior wall MI was induced in mice as previously described [22]. Briefly, animals were endotracheal intubated and ventilated with room air at 100 breaths per minute using a rodent ventilator (Harvard Apparatus). Sternotomy was performed and the proximal LAD was identified using a surgical microscope (M500, Leica Microsystems, Bannockburn, IL, USA) after retraction of the left atrium and ligated with 7–0 prolene. Blanching and dysfunction of the anterior wall verified LAD ligation. LAD ligation was performed by a surgeon blinded to the identity of the mice. After LAD ligation, the animals received 100 *μ*L of suspension of 1 × 10^5^ mouse MSCs through a tail vein injection. 24 hours after stem cell injection the animal was sacrificed for organ harvest.

### 3.4. Cell Preparation and Delivery

MSCs were prepared as previously described [23]. Six-week-old C57BL/6J mice were sacrificed and the hind limbs were removed. Their femurs were carefully cleaned of adherent soft tissue and bone marrow was flushed into a 50 mL falcon with flush medium (Alpha Medium with 2 g/L NaHCO_3_, 10% horse serum, 10% FBS, 1% L-Glutamine, 1% penicillin-streptomycin). The cells were filtered through a 70 *μ*m nylon mesh filter followed by centrifugation for 5 min at 260 g and washed with PBS. The washed cells were plated in flush medium and incubated at 37°C. Nonadherent cells were removed by replacing the medium after 24 h. The cells were cultured in a monolayer at 37°C and 5% CO_2_ and medium was refed every 3-4 days. When cells reached 80% confluence, adherent cells were detached after incubation with 0.05% trypsin and 2 mM EDTA (Invitrogen) for 5 min. Cells were depleted of CD45^+^, CD34^+^ cells by negative selection using 10 *μ*L per 10^6^ cells of each of the after primary PE-conjugated antibodies: mouse anti-rat CD45 (BD Biosciences, San Diego, CA, USA) and mouse anti-CD34 antibodies (Santa Cruz Biotechnology, Inc., Santa Cruz, CA, USA). PE-positive cells were negatively selected using the EasySep PE selection kit according to the manufacturer's instructions (Stem Cell Technologies, Vancouver, B.C., Canada). Cells were replaced in medium and were subsequently passaged until passage 6. Cells were labeled with Qtracker Cell Labeling Kit (Invitrogen) according to the manufacturer's instructions. 100,000 labeled MSCs suspended in 100 mL of PBS or 100 mL of PBS alone were infused via tail vein injection 24 h after myocardial infarction.

### 3.5. Specimen Preparation

Following sacrifice, the heart and lungs were removed. The organs were then separately embedded in optimal cutting temperature compound (OCT) (Tissue Tek, Ted Pella, Inc., Redding, CA) inside an aluminum foil mould. The entire mould is snap-frozen in liquid nitrogen for five minutes to reduce ice crystal formation. Following this, the mould assembly was removed from the liquid nitrogen bath and placed inside the cryomicrotome chamber to equalize the specimen temperature to that of the cryomicrotome. After three hours, the mould was removed and the frozen specimen mounted on the microtome stage and the slice thickness was set to 40 *μ*m. Heart specimens were prepared as described and imaged at 12.5x magnification, with a pixel size of 8.8 *μ*m. Lung specimens were prepared as described and imaged at 10x magnification, with a pixel size of 11 *μ*m.

## 4. Results

Detection of quantum dot-labeled cell clusters was compared to detection by a user in an infracted mouse heart. An operator manually detected clusters blinded to the algorithm results. It became quickly apparent that it was very difficult to visually detect single cell clusters that were often 1-2 pixels on a video screen. This required zoom and pan throughout the entire image. The operator detected *≈*80 clusters in 50 images, whereas the algorithm detected many more (*≈*250) clusters. The operator did not detect any cells that the algorithm missed, which would have indicated a false negative (FN). All cells detected by the algorithm were displayed and verified as cells by the user, giving no false positive (FP) detections. Hence, as best as we could determine, the algorithm had no FNs or FPs in these 50 images of heart tissue. Tissues having more autofluorescence (e.g., bone and gastrointestinal tract) could have detection errors.

**Figure 1:**
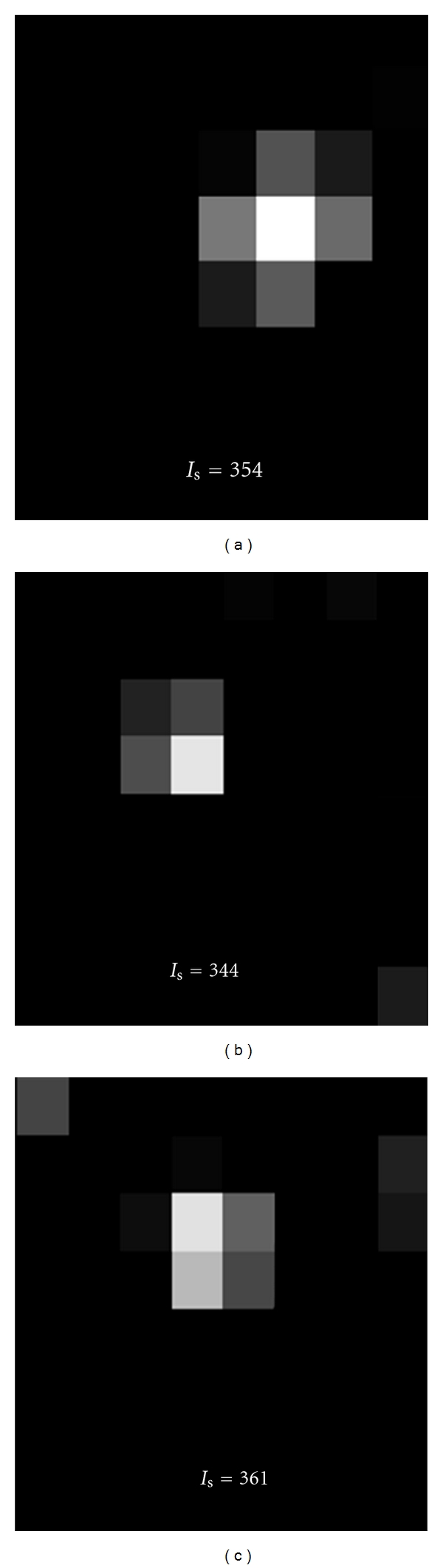
The appearance of single cells/microspheres is distorted by pixelation. The shape greatly depends upon the location of the microsphere with respect to the sampling grid of the CCD camera. Three separate single microspheres in OCT are shown with their corresponding integrated intensity. Although the images appear much different, the integrated intensity is nearly equal and within the 5% variation specified by the manufacturer.

**Figure 2:**
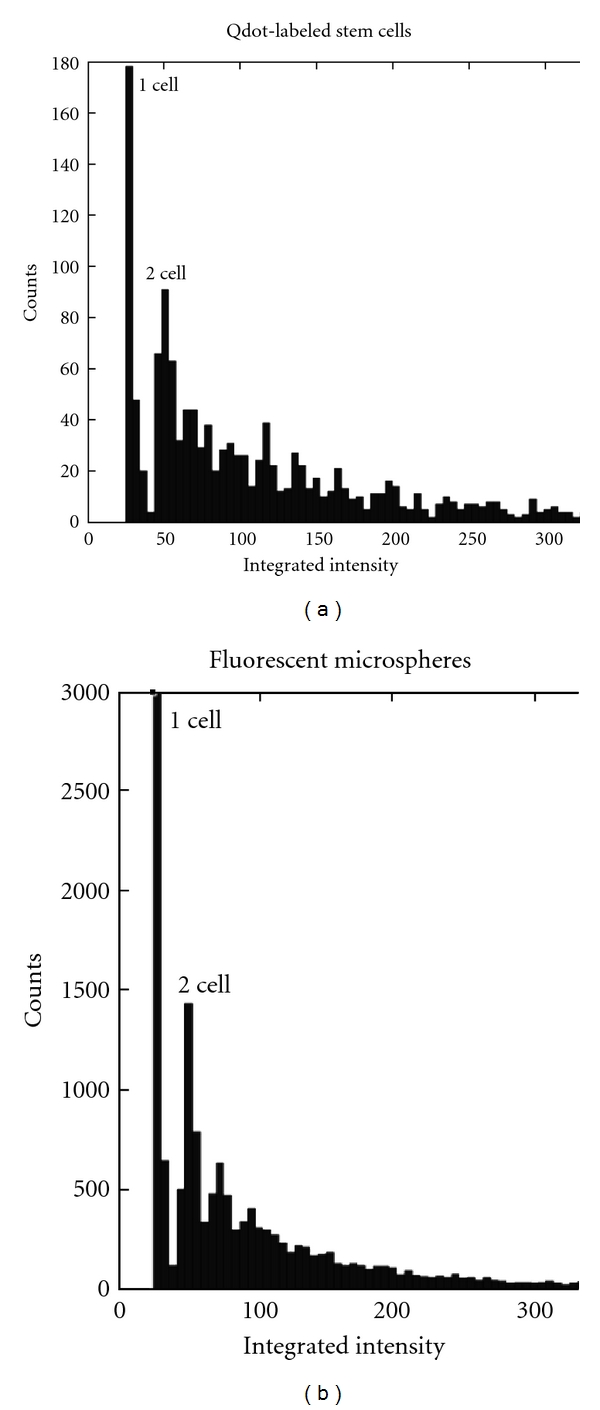
Histograms of the integrated intensity of clusters of fluorescently labeled cells and microspheres. Since cells/microspheres were dispersed, the largest peaks correspond to single cells/microspheres. Note that the next large peak occurs at 2 times the integrated intensity of the first peak, indicating two cells in a cluster. We use values in the “1 cell” peak to estimate parameters for subsequent processing of images.

We characterized microspheres and cell images in the OCT phantom. At the normal operating resolution, visual appearances of microspheres can change dramatically, depending upon the position of the microsphere with respect to the pixel grid (Figure 1). We are confident that all fluorescent clusters in Figure 1 are due to a single microsphere because the integrated intensity is very nearly equal (within 5%). Histograms of the integrated intensity of clusters showed a clear separation between single-microsphere/cell clusters and multiple-microsphere/cell clusters for both cells (Figure 2(a)) and microspheres (Figure 2(b)). Note that a clear, smaller peak corresponds to an intensity expected for two cells/microspheres.

**Figure 3:**
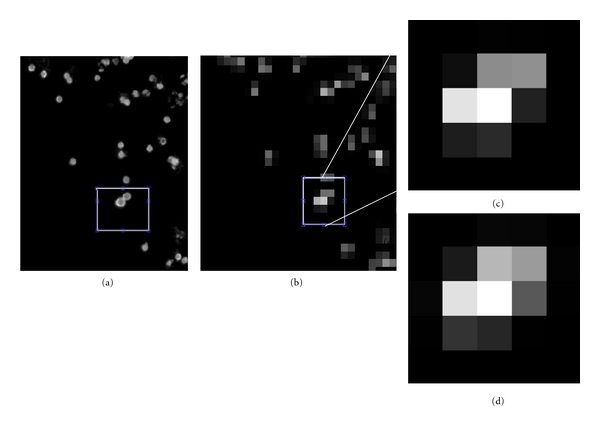
High-resolution images of microspheres are used to validate results of model-based processing on working-resolution images. High-resolution (63x) (a) and working-resolution (7x) images (b, c) of green fluorescent microspheres were obtained. There is a two-microsphere cluster in (a), which is visually ambiguous at the working resolution. The algorithm's result for a two-cell cluster in (d) visually matches the image in (c). Other numbers of microspheres resulted in a much poorer fit to data. Note that *≈*9 × 9 pixels in the high-resolution image correspond to 1 pixel in the low-resolution image and that the “grid” is not exactly maintained; that is, the 2D region in (a) only approximates regions in high-resolution images. The lower left microsphere in (a) is brighter than the other probably because it is closer to the surface.

**Figure 4:**
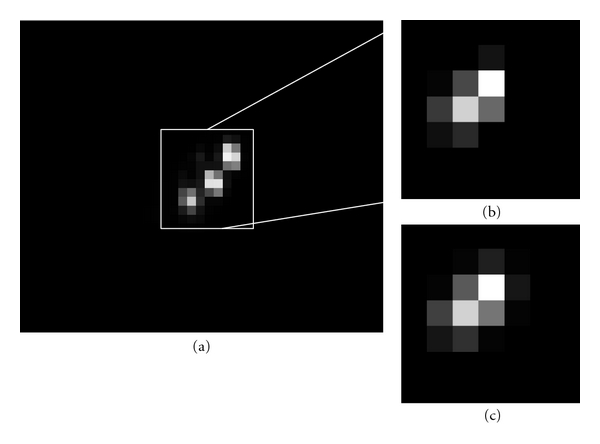
High-resolution images of fluorescently labeled MSCs are used to validate results of model-based processing on working-resolution images. High-resolution (20x) images (a) clearly show three cells. The corresponding low-resolution (7x) image (b) is visually ambiguous. The algorithm gives 3 cells, and the predicated image (c) well fits image data in (b). Note that *≈*3 × 3 pixels in the high-resolution image correspond to 1 pixel in the low-resolution image and that the “grid” is not exactly maintained. The 2D region in (a) only approximates the region in (b).

To test the ability of our software to correctly estimate the number of cells/microspheres in a cluster at normal operating resolution, we compared results to high-resolution images (Figures 3 and 4). Individual microspheres and cells were clearly visible in high-resolution (1.6 *μ*m pixels for microspheres and 5.4 *μ*m pixels for cells) images (Figure 3(a) and Figure 4(a), resp.). However, at normal operating resolution, images of the same microspheres or cells are joined into single connected clusters (Figures 3(b) and 4(b), resp.). The algorithm correctly estimated the number of cells/microspheres in the normal operating-resolution images (Figures 3(d) and 4(c), resp.). This process was repeated on many clusters as given below.

**Figure 5:**
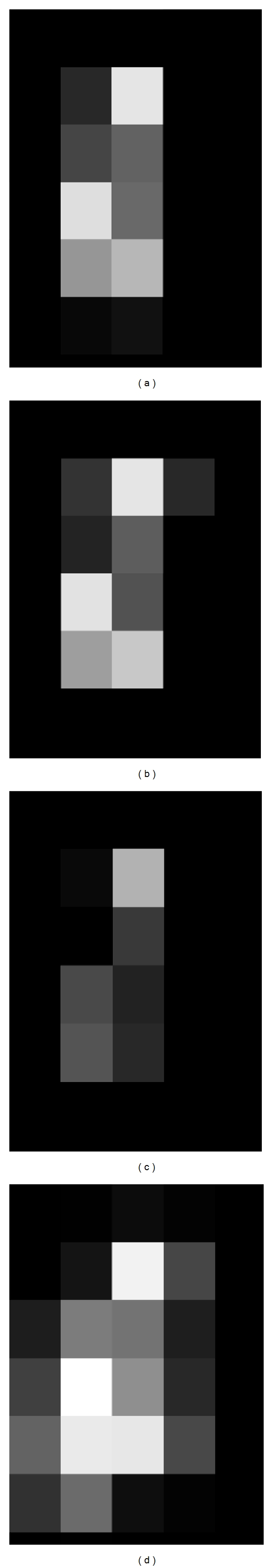
Uniqueness of the cell quantification model. A low-resolution cluster image (a) is shown compared to the best fit (b) containing four model cells. Results for three cells (c) and five cells (d) are clearly inferior. LSQ error equals 145, 231, and 386 gray values for images (b), (c), and (d), respectively.

The algorithm converged and gave unambiguous results. For example, in Figure 5, we modified the algorithm to test *≈*200 initial cell configurations to fit the detected cluster. In each case, the nonlinear optimization algorithm rapidly converged in 50–70 iterations, many fewer than the maximum (10,000). All of these restarts gave negligible differences in the final results. Results for 3, 4, and 5 cells are shown in Figure 5. Clearly, 4 is the best result both visually and quantitatively from the objective function. Essentially, unique solutions were found in 1000's of cells now tested. Almost all cell/microsphere positions changed <0.1 pixels with different initializations, and in the case of the best number of cells, results typically changed <0.001 pixels. We very rarely (*≈*0.1%) reached the maximum number of iterations, due to oscillatory behavior of the algorithm. In tests, we added a line in the code to determine the number of times that the objective function differed by less than 20%, a threshold giving similar visual results. In a run over 656 fluorescent clusters, we determined that only 1.2% of clusters gave such an “ambiguous” result. In many cases, the answer was correct, per the analysis below.

**Figure 6:**
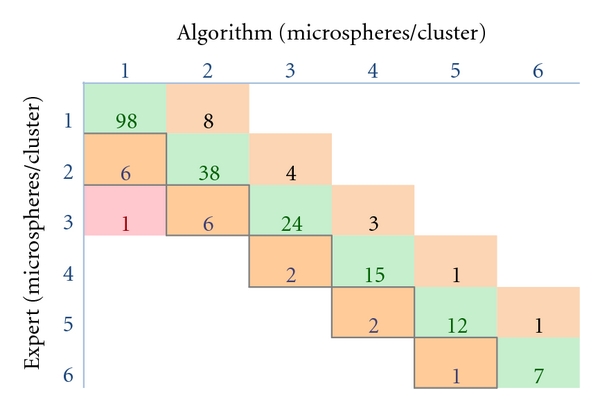
Contingency table analysis of algorithm accuracy on microsphere images. Microspheres/cluster were estimated using the algorithm on working-resolution images (7x). An expert visually determined the number of microspheres/cluster on corresponding high-resolution images (63x). Table entries are the number of times a result was obtained; for example, the algorithm and expert identified the same 2-cell clusters 38 times. In all cases but one, the algorithm was within ±1 microspheres. The total number of microspheres counted by the expert was 499 compared to 497 by the algorithm.

**Figure 7:**
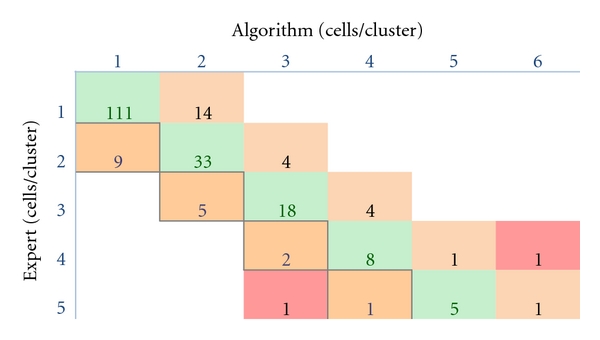
Contingency table analysis of algorithm accuracy on MSCs. In all cases but two, the algorithm was within ±1 cells. The total number of cells counted by the expert was 393 compared to 386 by the algorithm. See Figure 6 for details.

 We tested the quantification algorithm at the operating resolution against an expert using high-resolution images over a large numbers of microsphere (229) and cell (218) clusters. Model-based processing was first performed on a fluorescent microsphere phantom to evaluate the algorithm in the best case scenario with nearly identical fluorescent sources. A small percentage of clusters (*≈*5%) were visually ambiguous even at this high resolution, and these were excluded from our analysis. In Figure 6, we present a contingency table comparing the algorithm versus an expert reader. Perfect agreement consists of a diagonal in a contingency table. In all cases but one, the algorithm was within ±1 microspheres of the actual number present in a cluster (Figure 6). Model-based processing estimated the total number of microspheres present to be 497, compared to 499 counted by an expert, an error of only 0.4%. Fleiss' kappa score for the contingency table was 0.78 ± 0.04. When Fleiss' Kappa is 1, there is perfect agreement between the expert and the algorithm, with a value of zero corresponding to a random guess at the number of cells per cluster. Specificity in all cases was >0.95. Sensitivity varied depending on the number of microspheres per cluster, reaching a maximum at *n* = 1 of 0.85 and a minimum of 0.75 at *n* = 3. The mean was 0.80. Model-based processing was also performed on cells in OCT. In all cases but two, the algorithm was within ±1 cells of the actual number present in a cluster (Figure 7). The total number of cells counted by the expert was 393 compared to 386 by the algorithm, giving an error rate of only 1.7%. Fleiss' kappa was 0.68 ± 0.04. The specificity in all cases was >0.90. The sensitivity varied depending on the number of microspheres per cluster, reaching a maximum at *n* = 1 of 0.82 and a minimum of 0.625 at *n* = 5. The mean sensitivity was 0.71.

**Figure 8:**
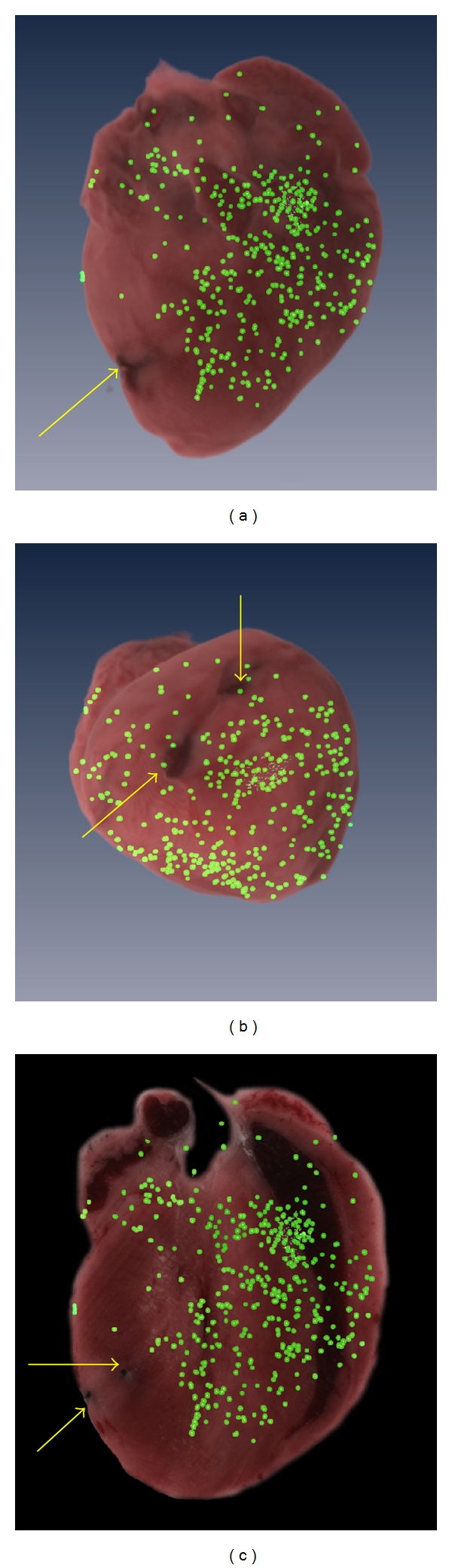
3D volume renderings of MSC locations in the heart. Model-based processing was used to perform segmentation, quantification, and localization of quantum dot-labeled MSC's in an infarcted heart. A 3D volume rendering of brightfield images of the heart is shown with a 3D volume rendering of MSC location 24 hours after a tail vein injection of 100,000 MSCs. MSCs were injected immediately following ligation of the LDA. MSC location was found to correspond to local blood flow in the infarcted heart. A long-axis (a) and short-axis (b) view of the heart shows cells located primarily on the right side of the heart. A corresponding long-axis 2D slice is shown along with a volume rendering of MSC location (c). In all images, the suture locations are visible (arrows).

 As an example application of our quantitative stem cell techniques, we show results from a study of cardiac stem cell therapy with MSCs. Following surgery to induce myocardial infarction in C57BL/6J male mice, we injected red quantum dot-labeled MSCs via the tail vein. At 24 hours postadministration, we reliably imaged MSCs. Cells were *≈*10x brighter than the autofluorescent background. Autofluorescence was minor, with a small amount of green autofluorescence in the infarct zone and relatively no autofluorescence elsewhere in the heart. Image results are shown in Figure 8. The reduction of blood flow to the left ventricle caused by LDA ligation resulted in increased concentration of MSCs on the right side of the heart (Figure 8). The relative distribution of MSCs in the heart is shown relative to the infarct location (Figure 8). Cells detected by the algorithm were displayed, verified by a user and found to contain only quantum dot-labeled cells. In total, 554 clusters were detected and 748 MSCs were estimated to be in the heart by model-based processing. We determined that 74% of clusters contained single cells, with no cluster containing more than 5 cells.

## 5. Discussion

We have developed a useful, well-validated method for detecting fluorescent clusters and determining the number of cells per cluster in cryo-image data. The numbers of cells or microspheres in a given cluster was verified through visual inspection, giving an excellent “gold standard” with which to compare. In the case of microspheres, in only one instance was the algorithm off by more than one microsphere. There was no substantive bias towards high or low numbers of microspheres within a cluster, and 497 and 499 microspheres were counted by the algorithm and manual inspection, respectively, giving a 99.6% accurate microsphere count. With quantum dot-labeled cells, there were more errors, but results were nevertheless quite impressive. Cell counts were 386 and 393, for algorithm and manual, respectively, giving a 98.3% accurate cell count. Remarkably, the algorithm was within ±1 cells of the actual number of cells in a cluster all but two times. The small decrease in accuracy with cells is most likely due to variability in the size and intensity of the cells. Cells were found to have a measured standard deviation of *≈*15% from the mean cell integrated intensity, corresponding to the 15% window used for FACS sorting. To achieve accuracy in cell and microsphere counting, drift and fluctuations in exciting light must be minimized, as determined in quality assurance tests.

Time savings with the algorithm are extraordinary. Our nonoptimized algorithm takes about 10 hours to analyze an entire mouse. We estimate that a similar manual analysis would require over 6 months.

A major innovation is the use of model-based processing to estimate the number of cells per cluster. Our approach fits the spatial intensity pattern of a cluster with a collection of cell models having known spatial extent and integrated intensity. Pixelation error does not affect quantification with such an approach. Pixelation of a cluster results in surrounding pixels of varying intensity, dependent upon the percentage of the fluorescent cluster in that pixel (Figure 1). A pixel containing a small fragment of a cluster may not be included by the low-threshold *T*_*L*_, resulting in a decrease in the recorded integrated intensity and volume of the cluster. This error in the recorded integrated intensity and volume affects the corresponding cluster histograms, resulting in an increased spread around the single-microsphere/cell cluster peak (Figure 2). Because the initial estimate for the number of cells/microspheres in a cluster is dependent upon the cluster's integrated intensity (4), this can lead to an error in the initial estimate. However, this will be corrected with the model-based estimate which uses a region two pixels larger in *x* and *y* than the thresholded component of the cluster. This includes pixels that may have been missed by the low threshold. Alternative cell segmentation approaches based on shape would necessarily require much higher resolution images impractical for our application.

MSC localization corresponded to reduction of blood flow caused by LDA ligation. Ligation of the LDA reduced blood flow to the front and bottom of the left ventricle, resulting in no fluorescently-labeled MSC's detected in the infarct zone (area surrounding the suture) in the heart (Figure 8). MSCs were found preferentially located in the myocardium surrounding the right ventricle (Figures 8(a) and 8(c)). All cell clusters found in the heart passed through the capillaries in the lung prior to entering the heart. For this reason, we expected the vast majority of cell clusters to contain single cells. While there were multiple-cell clusters present, clusters contained predominately single cells (74%), perhaps a result of filtering in the passage through the lung. Despite relatively small numbers of cells getting through the lung to the heart, there is evidence of cardiac recovery due to MSC therapy, probably due to a paracrine effect [24]. Cryo-imaging and quantitative stem cell analysis should greatly aid interpretation of future studies.

In conclusion, the accuracy of the model-based quantification software allows us to count cells over large regions of cryo-image volumes. The algorithms can be reliably applied on bright cells in homogeneous tissue. However, in highly autofluorescent regions (e.g., gastrointestinal tract and bones), very bright cells and/or improved autofluorescence rejection in image acquisition are required to ensure automated detection of all cells. Cryo-imaging allows us to capture high-resolution images with high sensitivity over large areas, while simultaneously imaging the tissue with color brightfield. Preliminary experiments demonstrate the effectiveness of cryo-imaging and model-based processing to detect, segment, and quantify stem cells in cell therapy. We believe that this new methodology will be useful in a myriad of studies on cell source (MSC, adipose stem cell, cord blood, and many more), cell treatments, dosing regimens, delivery method, and so forth, in a quantitative manner heretofore unavailable.

## Figures and Tables

**Table 1:** Pseudocode for the determination of model-based parameters and estimation of cell number. Histogram analysis of cluster volume and integrated intensity are used to estimate single cell clusters. Model parameters are determined from the estimated single cells. These parameters are used to estimate the number of cells in a given cluster.

	*Preprocessing and determination of model cell parameters *
	Load aligned, next image processed cryo-image stack
	Perform morphological reconstruction to remove background
	User interactively determines *T*_*R*/*G*_, *T*_*H*_, and *T*_*L*_
	Perform thresholding of ratio image
	Perform hysteresis thresholding
	Perform connected component analysis
	Display histogram of cluster volume
	User selects single cell volume range based on largest peak
	Display histogram of integrated intensity
	User selects single cell integrated intensity range based on largest peak
	For all clusters that are within the user defined integrated intensity and volume range
	Fit Gaussian with free parameters (*x*_*c*_, *y*_*c*_, *z*_*c*_), *I*_*t*_, and *σ* to each cluster
	Perform an average of estimated model parameters to determine *σ* and *I*_*t*_

	*Estimate number of cells per cluster*

	For a given cluster
	Estimate number of cells per cluster
	Test integer numbers of cells within the range
	Perform nonlinear optimization to place the model cells in the 3D positions that best fits the input image
	The number of cells that best fits the inputted image is chosen as the estimated number of cells in the cluster
